# Anti-malarial activity of the alkaloid, heptaphylline, and the furanocoumarin, imperatorin, from *Clausena anisata* against human *Plasmodium falciparum* malaria parasites: ex vivo trophozoitocidal, schizonticidal and gametocytocidal approach

**DOI:** 10.1186/s12936-023-04678-0

**Published:** 2023-09-09

**Authors:** Emmanuel Kofi Kumatia, Felix Kwame Zoiku, Alex Asase, Nguyen Huu Tung

**Affiliations:** 1 Department of Phytochemistry, Centre for Plant Medicine Research, P.O. Box 73, Mampong-Akuapem, Ghana; 2 Department of Quality Management, Centre for Plant Medicine Research, Mampong-Akuapem, Ghana; 3 Department of Epidemiology, College of Health Science, Noguchi Memorial Institute for Medical Research, University of Ghana, Accra, Ghana; 4 Plant Development Department, Centre for Plant Medicine Research, Mampong-Akuapem, Ghana; 5 Faculty of Pharmacy, Phenikaa University, Hanoi, 12116 Vietnam

**Keywords:** Malaria, Alkaloid, Coumarin, *Plasmodium*, Trophozoite, Schizont, Gametocyte, Cytotoxicity, Selectivity index

## Abstract

**Background:**

The erythrocytic stage of the life cycle of the malaria parasite, *Plasmodium falciparum*, consists of trophozoite, schizont and gametocyte stages in humans. Various anti-malarial agents target different stages of the parasite to produce treatment outcomes. This study reports on the stage-specific anti-malarial activity of heptaphylline and imperatorin against human *P. falciparum* in addition to their cytotoxicity and selectivity indices (SI).

**Methods:**

The compounds were isolated from *Clausena anisata* using column chromatography and their structures elucidated using NMR spectroscopy. The anti-malarial activity was determined by measuring the trophozoitocidal, schizonticidal and gametocytocidal activities of the compounds using the SYBR green assay. Cytotoxicity was evaluated using the tetrazolium-based colorimetric assay.

**Results:**

Heptaphylline and imperatorin produced trophozoitocidal, schizonticidal and gametocytocidal activities with IC_50_s of 1.57 (0.2317)–26.92 (0.3144) µM with those of artesunate (the standard drug) being 0.00024 (0.0036)–0.0070 (0.0013) µM. In the cytotoxicity assay, the compounds produced CC_50S_ greater than 350 µM and SI of 13.76–235.90. Also, the trophozoitocidal and schizonticidal activities of the compounds were more pronounced than their gametocytocidal activity. Imperatorin was 42.04% more trophozoitocidal than hepthaphyline. However, hepthaphyline has more schizonticidal and gametocytocidal properties than imperatorin.

**Conclusion:**

Heptaphylline and imperatorin are promising anti-malarial agents, since they possess potent anti-malarial activity with weak cytotoxicity on RBCs. However, imperatorin is a better anti-malarial prophylactic agent whereas heptaphylline is a better malaria treatment agent.

## Background

Malaria is a major public health challenge, which puts severe stress on health care systems, individuals and family incomes, especially in developing countries, as a result of hospitalizations and deaths [1, 2]. The *Plasmodium* parasite which causes malaria infects humans through the bite of an infected *Anopheles* mosquito and cause sickness. Over the years, efforts were made to treat, minimize and/or eradicate malaria infections by employing chemotherapeutic agents (e.g., artesunate or chloroquine) for treatment, prophylactic drugs (e.g., sulfadoxine–pyrimethamine) to prevent *Plasmodium falciparum* infections from developing into the disease, or prevention of parasite transmission to humans (e.g. using insecticide-treated nets to prevent mosquito bites [3–5]. These programmes have yielded great outcomes by reducing global malaria deaths from 1,817,000 in 2004 to 1,238,000 in 2010 and to 619,000 in 2022 [6, 7].

Despite, these successes, malaria is still on the rise as reported in the year 2021 [7]. Furthermore, the advent of insecticide resistance mosquitoes and artesunate resistance *Plasmodium* parasites puts the global efforts of malaria eradication at risk [8–10]. These challenges call for development of new drugs to fight malaria.

Medicinal plants are the greatest sources of diverse chemical entities, which provides treasure troves of resources for novel drug discovery. *Clausena anisata* is a plant used in traditional medicine to treat a broad range of human ailments, including infectious diseases such as schistosomiasis, leprosy, syphilis, gonorrhea, and malaria [11, 12]. Phytochemical investigations of the plant led to isolation and characterization of a host of compounds, which belong to various classes of phytochemicals such a carbazole alkaloids, simple coumarins, furanocoumarins and phytosterols [13–15]. Although, some biological activities were reported for some of the individual compounds, their anti-malarial activity against the different stages of the human *P. falciparum* is not known. This study, therefore, seeks to isolate, characterize and evaluate the anti-malarial activity of some of the constituents of *C. anisata* root bark against the trophozoite, schizont and gametocide stages of human *P. falciparum* malaria and to also determine their cytotoxicity and selectivity indices.

##  Materials and methods

### Chemicals and reagents

Petroleum ether (40–60 °C) and ethyl acetate were procured from Park Scientific Limited (Northampton, UK). Silica gel for column chromatography (40–60 μm particle size and 230 × 400 mesh size) and TLC plate (aluminum sheets coated silica gel 60F_254_) were also obtained from Sorbent Technologies (Atlanta, GA, USA) and Merck Chemicals (Damstadt, Germany) respectively. Ethanol (99%) was acquired from Midland Ghana Limited, Tema.

### Human *P. falciparum* parasite

Human *P. falciparum* parasite was obtained from a clinically ill patient, diagnosed of *P. falciparum* malaria at Damfa Health Centre, Accra, Ghana. The blood (1.0 mL) was aseptically collected into a sterile ACD tube with vacutainer needle and immediately transported on ice to the laboratory, after ethical clearance and consent of the patient were obtained.

### Processing of plant material and isolation of compounds

*C. anisata* was identified and harvested in March, 2020, from Ayikumah, (in the Shai Osudoku District in the Greater Accra Region, Ghana). Identity of the plant was confirmed by comparison with an old voucher sample (CPMR 5101) in the herbarium of the Centre for Plant Medicine Research (CPMR), Mampong-Akuapem. Ghana. The root was washed with potable water, debarked and the bark sun-dried for 6 days before being pulverized. The extraction and chromatography were performed by modification of a previous method [13]. Briefly, about 500 g of the powder was macerated twice with 70% ethanol (4 L × 2) for 4 days each. The extracts were combined, filtered and the ethanol distilled off in vacuum at 45 °C. The aqueous extract was partitioned with petroleum ether (PE), 0.5 L × 4, and ethyl acetate (EtOAc), 0.5 L × 4, to obtain petroleum ether, ethyl acetate and aqueous fractions. The dried ethyl acetate fraction (20.50 g) was fractionated over 450 g of silica gel using PE–EtOAc–EtOH solvent systems. The  elution was started with PE–EtOAc system with addition of EtOAc at 10% incremental rate until 100% EtOAc was attained. EtOAc–EtOH system was then introduced at10% incremental rate of EtOH until 100% EtOH was also attained. A total of 195 eluents were collected in an aliquot of about 200 mL. The eluents were then grouped into 8 major fractions (F1–F8) by comparison of their TLC chromatogram. F3 and F5 produced **CA1** and **CA2** respectively. **CA1** and **CA2** were then purified after repeated recrystallization in PE–EtOAc 5:1.

### NMR spectroscopy analysis

NMR spectra (1D and 2D) of the compounds were obtained using NMR machine (Bruker FT-NMR Spectrometer Avance TM 500 MHz, Germany) equipped with Bruker\TopSpin3.6.3, JJ 10 NMR data analysis software and the chemical shifts resonance (*δ*) measured in ppm [13].

### Evaluation of anti-malarial activity of the compounds using SYBR green assay

#### Preparation of assay solutions of the compounds

Approximately, 3 mg of each compound was added to 3 mL of 0.5% DMSO and vortexed to yield a stock solution of 1000 µg/mL. The solution was filtered with 0.2 μm pore size filter paper and diluted 10-fold with incomplete medium (ICM) to obtain a working solution of 100 µg/mL which was then taken through a 9-fold serial dilution with ICM to obtain 100, 50, 25, 12.5, 6.25, 3.13, 1.56, 0.78 and 0.39 µg/mL concentrations. The process was repeated for each compound to obtain the serially diluted solutions for the assay against the trophozoite, schizont and gametocyte stages, respectively.

#### Cultivation of the human *P. falciparum* parasite

Efficacy of the compounds against the 3 stages of the *P. falciparum* life cycle was assessed ex vivo using a modified method of Baker et al. [16]. Continuous asexual culture of *P. falciparum* was maintained in an atmosphere of 90% N_2_, 5% CO_2_, and 5% O_2_ at 37 °C in complete medium (CM) consisting of (10.44 g/L, RPMI 1640, 5.94 g/L, HEPES, 5 g/L, AlbuMAX II, 50 mg/L hypoxanthine, 2.1 g/L sodium bicarbonate). The procedure was repeated for the schizont and the gametocyte stages.

#### Cultivation of trophozoites of the human *P. falciparum* parasite

Parasite from the culture established above was then cultivated in O^+^ RBCs and maintained in the incubator with daily media changes until a parasitaemia of more than 5% trophozoites were obtained. The culture was then treated with 5% sorbitol to obtain a synchronized trophozoite stage. The parasite growth was monitored for 5 days by estimating percentage (%) parasitaemia using Giemsa-stained slides and light microscope (100× magnification) until parasitaemia of more than 5% was recorded. Parasite mix of 2% haematocrit with 1% parasitaemia were then prepared using uninfected blood to make a total of 10 mL in a CM for the plating [16].

#### Cultivation of schizonts of the human *P. falciparum* parasite

Firstly, the trophozoites were cultivated as described above and allowed to grow further to the schizonts at parasitaemia of > 8%. The parasite was then synchronized with 60% Percoll (30 mL Percoll + 17 mL RPMI + 3 mL 10XPBS), transferred into a 15 mL falcon tube and then spined at 600 g for 5 min. The supernatant was discarded and the pellet washed twice in incomplete RPMI. Final pellet was suspended in CM to create 20% hematocrit in a 15 mL falcon tube. Approximately 7 mL of 60% Percoll was measured into a fresh falcon tube and the parasite suspension (2.5 mL) was slowly layered onto the Percoll. The tube was then spined at 700 g for 10 min to obtain the schizonts as a distinct reddish-brown band which was transferred into a clean falcon tube and adjusted to 2% hematocrit by addition of 20 mL uninfected RBCs in CM before plating with the various compounds. Percoll density gradient centrifugation described in this experiment was used to enhance the purification of the schizonts [17].

#### Cultivation of gametocytes of the human *P. falciparum* parasite

Gametocyte culture was initiated as described by Foley et al. [18], with some modifications, at 4% haematocrit (200 µL of culture was measured into a new T-25 flask containing 5 mL CM). The culture was later transferred into a sterile T-75 culture flask after the parasitaemia growth was above 8%. The parasite was allowed to grow further until 9–10% parasitaemia was achieved by daily microscopy monitoring and media changes. The culture was then synchronized with 5% sorbitol to obtain only rings, which were treated with freshly prepared CM and 50 mM *N*-acetyl glucosamine. This was noted as day 0. The flask was flushed with gentle flow of gas and then capped tightly and returned into the incubator. Spent media was removed from day 1–4 without taking the blood cells. 50 mM *N*-acetyl glucosamine in CM was added to the flask and then gassed before returning into the incubator. On day 5, the spent media was taken out and only CM was added to the flask and then gassed before returning into the incubator. The culture flask was maintained by repeated gassing procedure up to day 14. Percentage gametocyte was estimated by counting number of gametocyte or 1000–1500 RBCs. Parasite suspension was made and later plated with the compounds.

#### Compound plating

Approximately, 100 µL of each nine dilutions (100, 50, 25, 12.5, 6.25, 3.13, 1.56, 0.78 and 0.39 µg/mL) were plated in duplicates in a 96 well coastal plate. Artesunate (15 ng/mL) was serially diluted and plated alongside as the standard control. About 100 µL of the parasite suspension with 2% haematocrit and 1% parasitaemia were added to each treated well starting from the 2nd to the 10th well. Furthermore, another 100 µL of the parasite suspension was added to each of the 11th well as a negative control. The procedure was repeated for the other compound. The plates were then, arranged in a modular chamber and gassed for 5 min with gas mixture of 90% N_2_, 5% CO_2_, and 5% O_2_ at 37 °C and then incubated at 37 °C for 72 h.

#### SYBR green assay

The trophozoitocidal, schizonticidal, gametocytocidal assays of the compounds were performed according to a previous method [19], with some modifications. After the 72 h, the plates were harvested and 100 µL lysing buffer containing SYBR green was added to each well with thorough and gentle mixing prior to incubation in the dark for 30 min. The excitation and emission wavelengths of the samples were then measured at 470 and 520 nm, respectively, using fluorometer (FLUOstar OPTIMA with software version 2.20). The concentration that inhibits 50% of the growth of *P. falciparum* parasite (IC_50_) was estimated from dose-response curves by non-linear regression analysis using Graph pad Prism version 7.0 Software (Graph Pad software, San Diego, CA, USA). The IC_50_s were later converted to molar concentration.

#### Cytotoxicity assay

The toxicity of the compounds to RBCs was evaluated using the tetrazolium-based colorimetric assay as described [20], with some modifications. Briefly, 100 µL of each compound with concentrations ranging from 6.25 to 100 µg/mL was measured into separate wells of a 96-well microtitre plate in duplicates. Uninfected red blood cells (100 µL) of 2% haematocrit were added to each well. The plates were then incubated at 37 °C for 72 h in a humidified incubator at 5% O_2_ and CO_2_. MTT solution (20 µL of 7.5 mg/mL in phosphate buffered saline) was added to each well and the plate incubated again for 2 h. Finally, Triton X-100 (150 µL) in acidified isopropanol was added to each well to dissolve any formazan formed. The plates were then kept in the dark for 24 h after which the optical densities were measured at 570 nm using a plate reader. The cytotoxicity concentration (CC_50_), which is the concentration of the compound required to kill 50% of the cells was then determined by plotting concentration of the compounds on x-axis and percentage of cell viability on y-axis with dose-response curves.

$$\text{Cell}\;\text{viability} \;(\%)= \frac{\text{A}{0}-\text{A}{1}}{\text{A}{0}} \times {100}$$

where A_0_ or A_1_ is the mean absorbance of wells with untreated (vehicle) or test wells.

### Selectivity index(SI)

The SI of the compounds was calculated as:

$$\text{SI}= \frac{\text{CC}{50}}{\text{IC}50}$$

### Statistical analysis

Each concentration of a respective compound was tested in duplicate and the IC_50_ results presented as ${\bar{\text{x}}}$ (SD) i.e., mean (standard deviations).

## Results

### Characterization of the compounds

#### Characterization of CA1

CA1 was obtained as bright yellow needle crystals (6.56 g). R_f_ = 0.603 (PE–EtOAc, 20:1); 0.400 (PE–CF, 5:1). ^1^H NMR (CDCl_3_, 500 MHz), δ 11.67 (1H, s, –NH), 9.95 (1H, s, –CHO), 8.22 (1H, s, H-4), 8.00 (1H, d, H-5, J = 7.05 Hz), 7.29 (1H, ddd, H-6, J = 3.28, 3.16 and 7.40 Hz), 7.43 (1H, m, H-7, J = 3.35 Hz), 7.99 (1H, dd, J = 7.7, 1.0 Hz, H-8), 3.67 (2H, d, H-1′, J = 6.90 Hz), 5.35 (2 H, t, H-2′, J = 5.93 Hz), 1.74 (3 H, s, H-4′), 1.80 (3 H, s, H-5′). ^13^ C NMR (CDCl_3_, 500 MHz), δ 195.44 (CHO), 157.86 (C-2), 140.16 (C-8a), 145.08 (C-9a), 115.49 (C-3), 125.90 (C-4), 119.82 (C-5), 125.36 (C-4b), 123.70 (C-6), 120.89 (C-7), 110.90 (C-8), 117.37 (C-4a), 109.08 (C-1), 25.75 (C-1′), 121.27 (C-2′), 134.21 (C-3′), 22.87 (C-4′), 18.15 (C-5′). These results correspond with the literature data for the carbazole alkaloid, heptaphylline [13]. Hence, CA1 was identified as heptaphylline.

### Characterization of CA2

CA2 was obtained as white spongy crystals (10.56 g). R_f_ = 0.286 (PE–CF, 3:1); 0.706 (PE–EtOAc, 4:1). ^1^H NMR (CDCl_3_, 500 MHz), δ 6.36 (1H, d, H-3; J = 9.6 Hz), 7.77 (1H, *d*, H-4, J = 9.4 Hz), 7.36 (1H, s, H-5), 7.69 (1H, d, H-2″, J = 8.00 Hz), 6.82 (1 H, d, H-3″, J = 6.84 Hz), 5.00 (2 H, d, H-1′, J = 7.10 Hz), 5.61 (1 H, t, H-2′, J = 7.35 Hz), 1.72 (3 H, s, H-4′), 1.74 (3 H, s, H-5′). ^13^ C NMR (CDCl_3_, 500 MHz), δ 160.57 (C-2), 114.70 (C-3), 143.83 (C-4), 116.51 (C-4a), 113.60 (C-5), 125.88 (C-6), 148.62 (C-7), 131.68 (C-8), 144.25 (C-8a), 146.60 (C-2″), 106.73 (C-3″), 70.10 (C-1′), 119.70 (C-2′), 139.70 C-3′), 25.83 (C-4′), 18.13 (C-5′). These data are in agreement with those reported for imperatorin [13]. CA2 was therefore, identified as imperatorin.

**Fig. 1:**
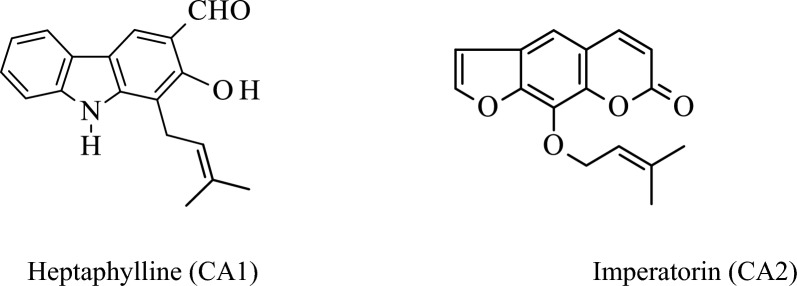
Chemical structures of the isolated compounds from *Clausena anisata* root bark

The chemical structures of the isolated compounds are given below in Fig. 1.

### Trophozoitocidal, schizonticidal and gametocytocidal activities of the compounds

**Fig. 2:**
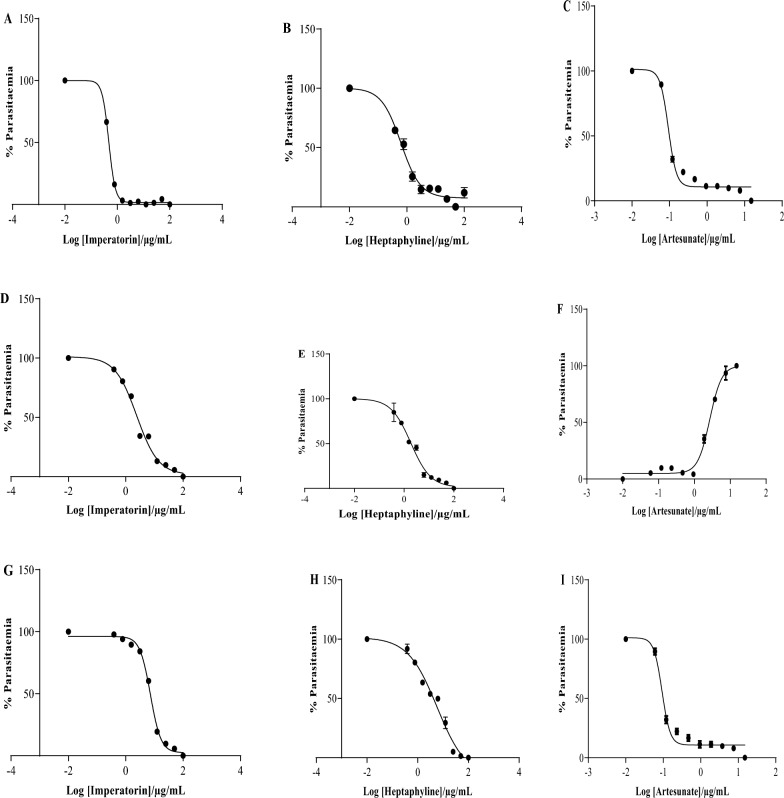
Ex vivo inhibition of: trophozoite (**A**–**C**); schizont (**D**–**F**) and gametocyte (**G**–**I**) stages of human *P. falciparum *by imperatorin, heptaphylline and artesunate. The shape of **F** reversed because the % parasitaemia inhibition was calculated from the highest to the lowest concentration instead of the reverse. However, the IC_50_ value is not affected by the shape of the graph

The IC_50_ curves (Fig. 2) and the IC_50_ values (Table 1) below shows the inhibitory effects of imperatorin, heptaphylline and artesunate on the various stages of the life cycle of human *P. falciparum* parasite ex vivo.

**Table 1:** The IC_50_ (SD) of the compounds on the inhibition of the various stages of *P. falciparum*

Compound	Trophozoite schizont gametocyte		
	IC_50_/µM	IC_50_/µM	IC_50_/µM
Imperatorin	1.57 (0.2317)	8.97 (0.8935)	26.92 (0.3144)
Heptaphylline	2.23 (0.0801)	6.71 (1.0094)	20.87 (0.1762)
Artesunate	0.00024 (0.0036)	0.0070 (0.0013)	0.00024 (0.00004)

The compounds strongly inhibited all the three stages (trophozoite, schizont and gametocyte) of the human *P. falciparum* malaria parasite. The minimum inhibitory concentration (IC_50_) of imperatorin or hepthaphyline against the trophozoite, schizont and gametocyte stages of the human *P. falciparum* were 1.57 (0.2317)–26.92 (0.3144) µM and 2.23 (0.0801)–20.87 (0.1762) µM respectively (Table 1). The furanocoumarin, imperatorin, was 42.04% more active against the trophozoites of the human *P. falciparum* malaria parasite than the carbazole alkaloid, hepthaphyline. Apart from the trophozoite stage, heptaphylline produced the lowest IC_50_ against the schizonts and the gametocytes compared to imperatorin. Hence, heptaphylline is a better schizonticidal (33.68% higher) and gametocytocidal (31.15% higher) agent than imperatorin.

Although the compounds produced very good inhibitory action on the various stages of the malaria disease, they were most active against the trophozoites followed by the schizonts. The least activity of the compounds was observed on the gametocytes (Table 1).

### Imperatorin and heptaphylline are weakly cytotoxic against human RBCs

The CC_50_ and the SI of the compounds on the trophozoite, schizont and gametocyte stages of the parasite are reported below (Table 2).

**Table 2:** CC_50_ and SI of the compounds against the various stages of human *P. falciparum*

Compound	CC_50_/µM	SI of the compounds on various stages of human *P. falciparum*		
		Trophozoite	Schizont	Gametocyte
Imperatorin	370.37	235.90	41.28	13.76
Heptaphylline	358.42	160.73	53.41	17.17
Artesunate	Nd	Nd	Nd	Nd

*Nd* not determined

## Discussion

In humans, the malaria parasite undergoes development in three stages in the RBCs. Liver form schizonts which envelops thousands of merozoites developed from sporozoites inoculated into the human skin through a bite of an infected *Anopheles* mosquito, disintegrates and release the merozoites into the blood stream where they invade RBCs to commence the intraerythrocytic asexual life cycle phase of the parasite [21, 22]. The trophozoite stage is the first phase of this process where the merozoites invades the RBCs and occupy a small portion of its food vacuole in a form of a small round ring (ring or early trophozoite stage) and feed on the hemoglobin and protein content of the RBCs and grow bigger to occupy most of the RBCs’ cytoplasm (trophozoite or late trophozoite) stage. The second stage is called the schizont stage. At this stage, the trophozoite divides into several small daughter cells called merozoites. The mature exo-erythrocytic schizonts erupts and release the merozoites along with RBCs and parasite remains (such as hemozoin and glycophosphatidylinositol) into the bloodstream [23]. The merozoites then invades healthy RBCs and the process continues [23]. In the gametocyte stage, the merozoites in some cells developed into male and female sexual forms called gametocytes, instead of undergoing asexual replication, which circulate in the blood where they are ingested by a vector mosquito during a blood meal, the male and the female gametocytes then meet in the mosquito and undergo various development to become sporozoites needed for human infection [23].

The trophozoite and gametocyte stages of the parasite do not manifest the malaria ailment in the human host. The clinical form of the disease is produced by the schizont stage of the parasite, when the RBCs erupt to release merozoites and together with parasite products, which activate the host immune response to produce cytokines, free radicals and additional cellular constituents, which are accountable for the symptoms such as fever, perspirations, chills, fatigue, and other systemic injuries, which eventually lead to the severe forms of the disease and death [24–26]. Thus, in order to achieve the best treatment outcome and relief the symptoms of malaria, the anti-malarial agent must be very active against the schizont stage of the parasite. All the tested compounds were very effective against the schizont stage of the clinically isolated *P. falciparum*. Therefore, they are good treatment substances for malaria. The compound that will produce the best treatment outcome against clinical malaria among the tested compound is heptaphylline. Since, it was the most effective against the schizont stage of the parasite than imperatorin.

Furthermore, the gametocyte stage is responsible for perpetuating the survival/life cycle and reinfection of the malaria parasite from humans to mosquito and back to humans. The compounds also effectively inhibited the gametocyte stage of the *P. falciparum* malaria parasite. This shows that the compounds and artesunate can be used to effectively prevent the transmission of human *P. falciparum* malaria parasite to the mosquito vector, thereby disrupting the life cycle of the parasite. The order of this effect was discussed in the results of the gametocyte stage.

Finally, the trophozoite stage of the *P. falciparum* parasite is the initiation stage of malaria. Thus, if the trophozoites are destroyed, then the clinical manifestations of malaria will not occur. Since, the compounds inhibited the trophozoite stage of *P. falciparum* parasite better than all other stages, indicates that the tested compounds can prevent malaria infection and transmission.

Artesunate is a semisynthetic analog of artemisinin (a plant-based anti-malarial drug extracted from *Artemisia annua*). Artesunate is produced by chemically incorporating hydrophilic group into the structure of artemisinin; hence making artesunate more efficacious drug than its parent drug, artemisinin and other artemisinin derivatives [8]. This suggest that modification of the structure of imperatorin and heptaphylline may also increase their efficacy as anti-malarial agents. It was reported that imperatorin was inactive as anti-plasmodial agent against *P. falciparum* D6 and W6 clones, respectively [27]. Heptaphylline was also reported to demonstrate antiplasmodial activity against *P. falciparum* K1 multi-drug resistance strain with IC_50_ of 3.2–6.4 µg/mL [28]. However, this is the first report on the stage-specific anti-malarial activity of both imperatorin and heptaphylline against the clinical isolate of the  human malaria parasite.

In the cytotoxic assay, both imperatorin and heptaphylline produced CC_50_ values which were greater than 350 µM against human RBCs with percent cell survival of greater than 50%. This indicates that the compounds were safe or weakly toxic to human RBCs, even at the highest concentration of 350 µM. Since, heptaphylline and imperatorin are nontoxic to the RBCs indicates that the anti-malarial activity of these compounds are intrinsic i.e., the compounds move across the membranes of the RBCs to kill the *Plasmodium* parasites in them without damaging the RBCs themselves.

The selectivity index (SI) was determined as the ratio of the 50% cytotoxic concentration of the compounds against the RBCs (CC_50_^RBCs^) to the minimum inhibitory concentration (IC_50_^Pf^) of the compound against *P. falciparum*. De Souza et al. [29], reported that SI values > 10 indicates safety since, the concentration at which the test compound is effective against the parasite is smaller than the concentration at which the same compound is toxic to the human cell. In this study, the compounds produced SI > 10 (SI = 13.736–211.864 for imperatorin and 17.152–160.771 for heptaphylline) respectively against the trophozoites, schizonts and gametocytes of *P. falciparum*. This indicates that both imperatorin and heptaphylline are nontoxic to human RBCs at all stages of malarial treatment. Imperatorin was also reported to be noncytotoxic on African monkey kidney fibroblast cell line (VERO) with an IC_50_ of 28.7 µg/mL in the XTT assay [27].

## Conclusion

This study has shown, for the first time, that the carbazole alkaloid, heptaphylline and the furanocoumarin, imperatorin possessed potent anti-malarial activity as a result of their trophozoitocidal, schizonticidal and gametocytocidal actions on the human *P. falciparum* parasite ex vivo. Furthermore, the results also indicated that both heptaphylline and imperatorin are weakly toxic to human RBCs with high selectivity indices against the trophozoites, schizonts and gametocytes of the human *P. falciparum* parasite. Heptaphylline and imperatorin are, therefore, promising anti-malarial agents to be considered for development into the next generation of anti-malarial drugs.

## Data Availability

The datasets produced and analysed during this study in addition to the isolated compounds can be obtained from the corresponding author upon reasonable request.

## Abbreviations

PE: Petroleum ether
EtOAc: Ethyl acetate
EtOH: Ethanol
CF: Chloroform
CM: Complete medium
ICM: Incomplete medium
SI: Selectivity index
